# Cu and Ni Co-sputtered heteroatomic thin film for enhanced nonenzymatic glucose detection

**DOI:** 10.1038/s41598-022-11563-4

**Published:** 2022-05-07

**Authors:** Brianna Barbee, Baleeswaraiah Muchharla, Adetayo Adedeji, Abdennaceur Karoui, Kishor Kumar Sadasivuni, Mizaj Shabil Sha, Aboubakr M. Abdullah, Gymama Slaughter, Bijandra Kumar

**Affiliations:** 1 Department of Mathematics, Computer Science and Engineering Technology, Elizabeth City State University, Elizabeth City, NC 27909 USA; 2 Department of Natural Sciences, Elizabeth City State University, Elizabeth City, NC 27909 USA; 3 Center for Research Excellence in Science and Technology (CREST), Department of Mathematics and Physics, North Carolina Central University, Durham, NC 27707 USA; 4 Center for Advanced Materials, Qatar University, 2713 Doha, Qatar; 5 Center for Bioelectronics, Old Dominion University, 4211 Monarch Way, Norfolk, VA 23508 USA

**Keywords:** Electrocatalysis, Biosensors

## Abstract

In this work, we report a wafer-scale and chemical-free fabrication of nickel (Ni) and copper (Cu) heteroatomic Cu–Ni thin films using RF magnetron sputtering technique for non-enzymatic glucose sensing application. The as-prepared wafer-scale Cu–Ni thin films exhibits excellent electrocatalytic activity toward glucose oxidation with a 1.86 μM detection limit in the range of 0.01 mM to 20 mM range. The Cu–Ni film shows 1.3- and 5.4-times higher glucose oxidation activity in comparison to the Cu and Ni electrodes, respectively. The improved electrocatalytic activity is attributed to the synergistic effect of the bimetallic catalyst and high density of grain boundaries. The Cu–Ni electrodes also possessed excellent anti-interference characteristics. These results indicate that Cu–Ni heteroatomic thin film can be a potential candidate for the development of non-enzymatic glucose biosensor because of its chemical free synthesis, excellent reproducibility, reusability, and long-term stability.

## Introduction

Diabetes is one of the main chronic diseases afflicting millions of people in the twenty-first century. Diabetes is the result of excessive amounts of sugar in blood. Glucose monitoring is essential because high or low blood-glucose levels in the body lead to hyperglycaemia or hypoglycaemia, respectively^1^. Continuous blood glucose monitoring is critical to detect and treat patients in the early stages of diabetes. Thus far, various techniques such as spectrometry^2–4^, fluorescence^5–7^, chemiluminescence^8–10^ and electrochemistry^11–14^ have been successfully developed to detect glucose concentration. Among the available detection methods, electrochemical method of sensing glucose poses several advantages such as low-cost, simple operation, and rapid detection progress. A significant research effort has been devoted for the development of enzyme-based glucose sensors over the years^15–20^. However, enzyme-based glucose sensors require complicated enzyme purification procedures and their high fabrication cost, lack of long-term stability due to enzyme denaturation, and low sensitivity owing to indirect electron transfer^21–23^. Non-enzymatic electrochemical biosensors offer the direct electrocatalytic detection and cost-effective fabrication, high stability, and repeatability^24,25^.

The performance of the non-enzymatic glucose sensors fundamentally relies on the properties and nature of the electrodes. Of the various factors previously highlighted for establishing a high-performance non-enzymatic glucose sensor, an attractive material choice and nanostructure optimization serves as an effective strategy to enhance the electrode surface area, electrocatalytic activity, and effective electron transfer from electrocatalyst to conductive electrode substrate^26^. Therefore, noble metal nanostructure (e.g., Pt, Au and Ag)^27–29^, metal oxides (e.g., CuO, NiO, ZnO, Co_3_O_4_, WO_3_, MnO_2_, Fe_2_O_3_ and others)^30,31^ are primarily examined for non-enzymatic glucose sensors. To enhance the performance of the electrodes, metals or metal oxides-based nanocomposite prepared via integrating these elements with carbon nanomaterials (e.g., CuO/Graphene, Pt/carbon nanotubes), hybrid metal oxides (e.g., CuO/NiO) and metal alloys/composites (e.g., Pt–Pd, Pt–Au) have been examined^26^. Bimetallic electrodes exhibit improved performance, compared to their corresponding single metallic catalysts, due to alteration in coordination number of atoms and modified electronic structure to induced local strains. Noble metals have also been combined with non-noble metals to reduce the cost of fabrication without compromising the performance of the electrode. In this aspect, Ni is an ideal candidate due to its remarkable selectivity in catalytic oxidation of glucose with negligible effect of adsorbed chlorine ions or other oxidized intermediates. For example, the combination of Au–Ni core–shell structure exhibits superior performance i.e., fast response (~ 3 s), wide linear range for the glucose detection (0.5 to 10 mmol L^−1^) low detection limit (15.7 μM), but poor sensitivity (3.17 μA cm^−2^ mM^−1^)^32^. In another study, PtNi^33^ alloy-graphene electrodes also exhibit glucose detection capability in a wide concertation range (0.5–15 mM), and low detection limit (~ 16 μM) with a high sensitivity of 24.03 μA mM^−1^ cm^−2^. In contrast, the combination of Pd and Ni yielded an ultralow detection limit of 0.15 μM with a narrow range of 0.5–1.1 mM glucose^34^. In most of the studies, the process of materials synthesis involves usage of hazardous chemicals and is difficult to scale-up fabrication. Additionally, binders (e.g., Nafion) are essential to fabricate the sensors on conductive substrate, however, they inevitably reduce catalytic activity and reproducibility via blocking active sites. Other efforts such as electrochemical anodization has been proposed to overcome these issues, however, scalability and the introduction of secondary metal atoms in a chemical free process remain a big challenge.

This work implemented a sputtering technique to synthesize electrocatalyst thin films at a wafer-scale without using any hazardous chemicals for non-enzymatic glucose detection application. Cu and Ni co-sputtered (Cu–Ni) thin films have been grown on the Si substrate by using the RF magnetron sputtering technique. The performance of the Cu–Ni electrodes was examined by evaluating sensitivity, selectivity, reproducibility and stability through electrochemical experiments in 0.1 M NaOH (pH 13). A comparison with Cu and Ni electrodes demonstrates that Cu and Ni atoms work synergistically resulting in significantly superior electrocatalytic ability towards glucose oxidation (Cu–Ni ~ 3.1, Cu ~ 2.41, Ni ~ 0.57 mA cm^−2^ at an applied operating potential of 0.65 V). Moreover, Cu–Ni electrodes exhibited a wider linear range (0.01–2.0 mM) of glucose concentration with high stability (retaining 98% of oxidation signal after 100 cycles) and reproducibility (retaining 91% of oxidation signal after 75 days) without compromising the selectivity. This scalable and chemical-free process offers excellent performance of Cu–Ni and suggest their potential application in the field of biosensing particularly for glucose detection.

## Results

### Characterization of the Cu–Ni thin film electrode

**Figure 1:**
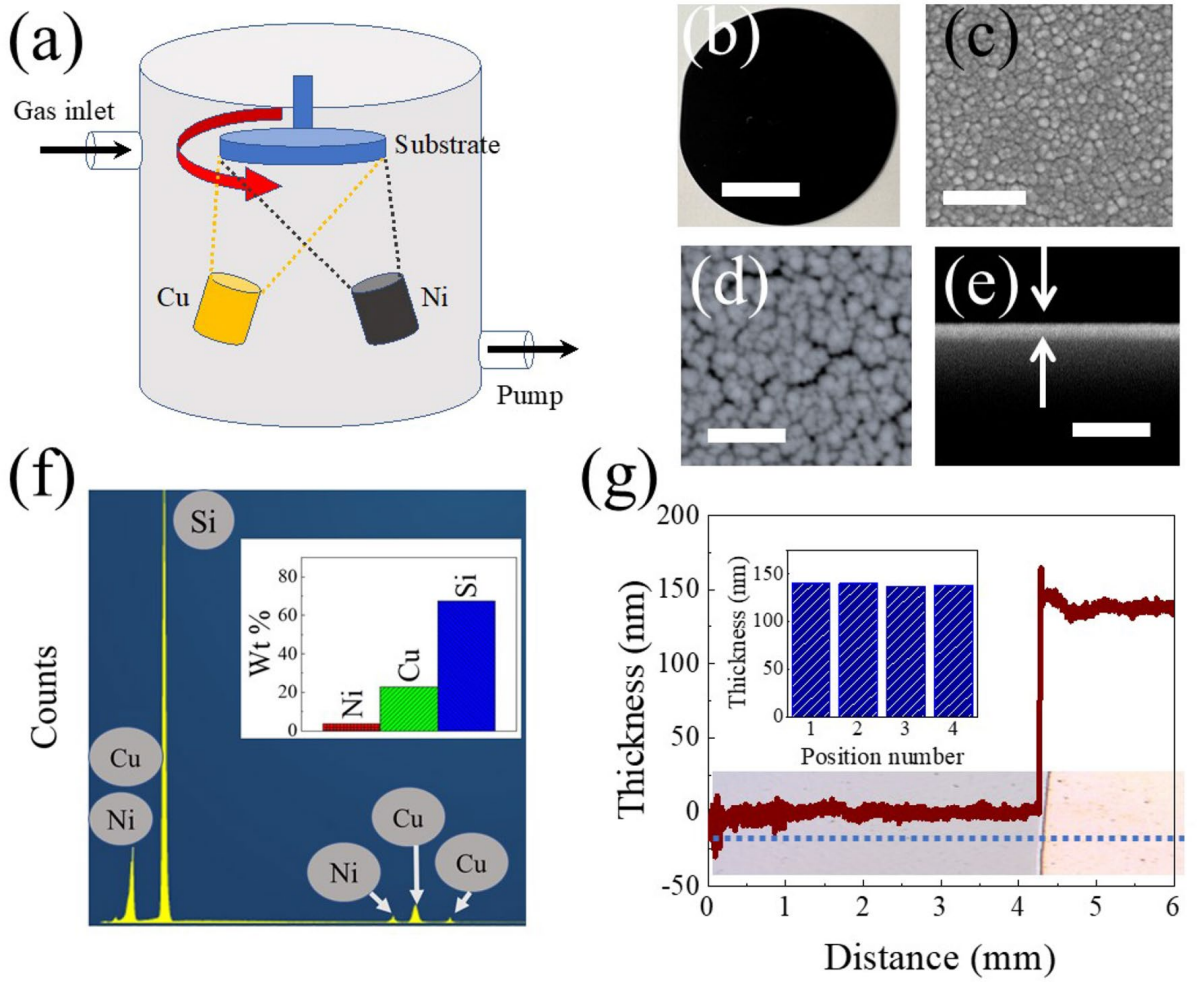
Characterization of Cu–Ni thin film: (**a**) Schematic illustration of the sputtering deposition chamber, (**b**) optical image of thin film deposited on the wafer (scale bar is 2 cm), (**c**, **d**) SEM micrographs of Cu–Ni thin film with a scale bar on the image (**c**) is 200 nm and on the image (**d**) is 100 nm, (**e**) cross-sectional SEM micrograph of Cu–Ni thin film with sale bar 1 µm, (**f**) EDS spectrum of Cu–Ni thin film with inset displays wt% of elements from EDS results, and (**g**) thickness profile of Cu–Ni thin film and bar graph in the inset provides the thickness of Cu–Ni thin film measured at various positions on the thin film.

Wafer (diameter 2.0 inch) scale Cu–Ni electrodes were prepared by RF sputtering thin film deposition methods (see “Experimental methods” section). Figure 1a shows the schematic illustration of a thin film deposition chamber depicting Cu and Ni targets powered on simultaneously to realize co-sputtered Cu–Ni thin film. Optical image of the Cu–Ni thin film deposited on 2.5-inch Si wafer is displayed in Fig. 1b. The scanning electron microscopy (SEM) micrograph of the deposited Cu–Ni thin film Fig. 1c shows the morphology of the uniformly formed thin film with tightly packed nano grains. High magnification SEM micrograph further confirms the presence of tightly packed grains with varying sizes of 10 nm to 20 nm forming multiple grain boundaries Fig. 1d. Such grain boundaries are provided highly electrochemical active sites for different electrochemical reactions such as CO_2_ electrochemical conversion^35^. The cross-section of Cu–Ni thin film displays the contrast to the substrate and confirms the grown homogeneous film of ~ 150 nm thickness (Fig. 1e). In addition, the energy-dispersive X-ray spectroscopy (EDS) confirms the presence of only Cu and Ni in the thin film Fig. 1f. The signal for Si atoms is obtained due to substrate. The EDS results confirm the purity of the thin film as other atoms were not detected. A Cu/Ni atomic ratio of about 5.5/1 was observed from EDS analysis. The thickness of the films was also measured at different points of Si wafer, and these results further confirm the growth of homogeneous Cu–Ni thin film at wafer-scale as minor variation (~ 4%) in height measured at different points was observed Fig. 1g.

### Electrochemical activity of Cu–Ni electrode for glucose sensing

**Figure 2:**
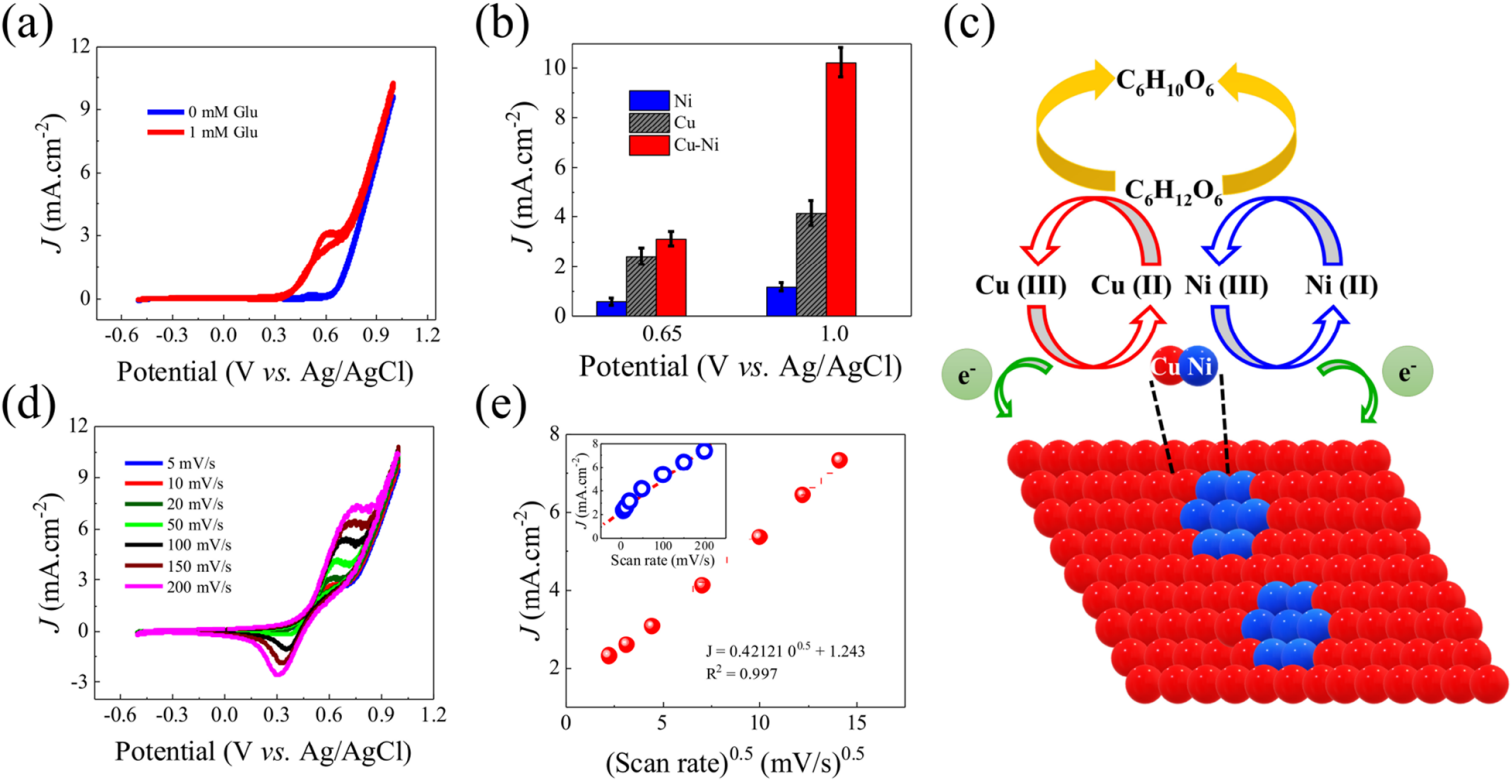
Glucose sensing characteristic of electrodes: (**a**) CV of Cu–Ni thin film electrode in the presence of 1 mM glucose concentration compared with the CV of no glucose concentration, (**b**) comparison of the glucose oxidation current at 0.65 and 1.0 V for three electrodes, (**c**) Schematic illustration for electrocatalysis of Cu–Ni for glucose oxidation, (**d**) CVs of Cu–Ni thin film electrode at different scan rates from 5 to 200 mV s^−1^ in 0.1 M NaOH with 1.0 mM glucose, (**e**) glucose oxidation current versus square root of scan rate fitted with a straight line and inset of (**e**) shows oxidation current versus scan rate for comparison.

Glucose sensing capabilities of Cu–Ni thin films were characterized by performing cyclic voltammetry (CV) experiments via sweeping potential between − 0.5 and 1.0 V with the scan rate of 20 mV s^−1^ in the presence and absence of glucose (Fig. 2a) in 0.1 M NaOH (pH 13) electrolyte. All potentials are reported with respect to the Ag/AgCl (saturated KCl) electrodes. In the absence of glucose, an exceedingly small oxidation peak around ~ 0.5 V was observed. This peak may be attributed to the oxidation of Cu/Ni atoms during the electrochemical experiment in NaOH solution. The CV collected in the presence of 1 mM glucose concentration, resulted in significant oxidation current in the region of 0.5–0.7 V. This is directly associated with the glucose oxidation reaction^12,24,36,37^. To uncover, if the observed performance is associated with the individual Cu or Ni atoms or whether the co-sputtered Cu–Ni thin film exhibits a synergistic effect, we performed glucose-sensing experiments for Cu- and Ni-based electrodes under similar experimental conditions and collected CVs (Figs. S1 and S2). We compare the glucose oxidation current at 0.65 V, normally used as an operational potential for Cu and Ni electrodes, and 1.0 V (Fig. 2b). At 1.0 V, oxidation of adsorbed hydroxyl species occurs, therefore a slight change in oxidation current is observed after glucose addition to the Cu–Ni electrode. Interestingly, the Cu–Ni electrode shows more than one order of magnitude higher current density than the pristine Ni electrode at 0.65 V potentials (glucose oxidation reaction potential). We also observed significantly higher oxidation currents for Cu–Ni comparison to the Ni (5 times higher) and Cu (2.3 times higher) electrode at 1.0 V potential, respectively. Electrochemical impedance spectroscopy (EIS) (Fig. S7.) evidence that the charge transfer resistance *R*_ct_ (26.15 Ω) and equivalent series resistance *R*_e_ (6 Ω) of Cu–Ni thin film electrodes are significantly smaller than those of the individual bulk Cu (*R*_ct_ ~ 47 Ω, *R*_e_ ~ 12 Ω) and sputtered Ni (*R*_ct_ ~ 213 Ω, *R*_e_ ~ 50 Ω) thin film electrodes. Thus, fast charge transfers due to minimum *R*_ct_ results in the superior performance Cu–Ni electrode among all studied electrodes. These results suggest that Cu–Ni co-sputtered thin film exhibits synergistic activity toward glucose, therefore resulting in superior performance. When Cu and Ni work synergistically for glucose sensing, multiple electrochemical reactions occur simultaneously^37^. A schematic illustration for electrocatalysis of Cu–Ni thin film electrode for glucose oxidation is displayed in Fig. 2c. Initially, Cu and Ni atoms are oxidized and then participate in glucose oxidation reactions as expressed in reaction (1) and (2)^37^.

1 $${\text{NiO }} + {\text{ CuO }} + {\text{ 2OH}}^{ - } \to {\text{ NiO}}\left( {{\text{OH}}} \right) \, + {\text{ CuO}}\left( {{\text{OH}}} \right) \, + {\text{ 2e}}^{ - }$$

2 $${\text{NiO}}\left( {{\text{OH}}} \right) \, + {\text{ CuO}}\left( {{\text{OH}}} \right) \, + {\text{ 2Glucose }} \to {\text{ NiO }} + {\text{ CuO }} + { 2}\;{\text{gluconolactone}}$$

In addition, CV experiments were performed at various scan rates (5, 10, 20, 50, 100, 150, and 200 mV s^−1^), and the magnitudes of anodic peaks were plotted for the square root of scan rates (Fig. 2d,e). The anode and cathode peaks gradually increase with respect to the change in the scan rates (Fig. 2d). A positive shift in anode peak and a negative one in cathode peak with respect to the increment in scan rate have also been observed. Here it should be noted that a relation between the square root of the scanning rate and magnitudes of anode/cathode peak provides insight into the reaction mechanism^30,37–39^. The CV curves of Cu (Fig. S3) and Ni (Fig. S4) electrodes with varying scan rates are provided in supporting information for comparison. The linear regression equation of oxidation peak current is *j* (mA cm^−2^) = 0.421*x* + 1.243 (*R*^2^—0.997) where *x* stands for ν^1/2^ (Fig. 2e) indicating linear relation between the square root of scan rate and oxidation current, confirming that the glucose oxidation at Cu–Ni electrodes is a diffusion-controlled and fast reaction kinetics reaction process. The diffusion coefficient (*D*) for the transfer of glucose at the time of electrocatalysis is estimated using the Randles–Sevcik equation^40^ as shown below [Eq. (3)]

3 $$I_{p} = 2.69 \times 10^{5} n^{3/2} AD^{1/2} C \nu^{1/2}$$

where *I*_*p*_ is the peak current density in amperes, *n* is the electron transfer number (usually 1), *A* is the electrode surface geometrical area (in cm^−2^), *C* is the bulk concentration (in mol cm^−3^), and $\nu$ is the scan rate (in V s^−1^) and *D* is the diffusion coefficient. Diffusion coefficients was determined from the slopes of *I*_*p*_ vs $\nu^{1/2}$, for Cu–Ni (Fig. 2e), Cu (Fig. S5) and Ni (Fig. S6) electrodes, respectively. The obtained diffusion coefficient values of Cu–Ni, Cu and Ni electrodes are 5.82, 0.05 and 0.001 cm^2^ s^−1^, respectively. This is further evidence that co-sputtered Cu–Ni thin film electrode favors higher glucose transfer resulting superior performance than individual Cu and Ni electrodes.

**Figure 3:**
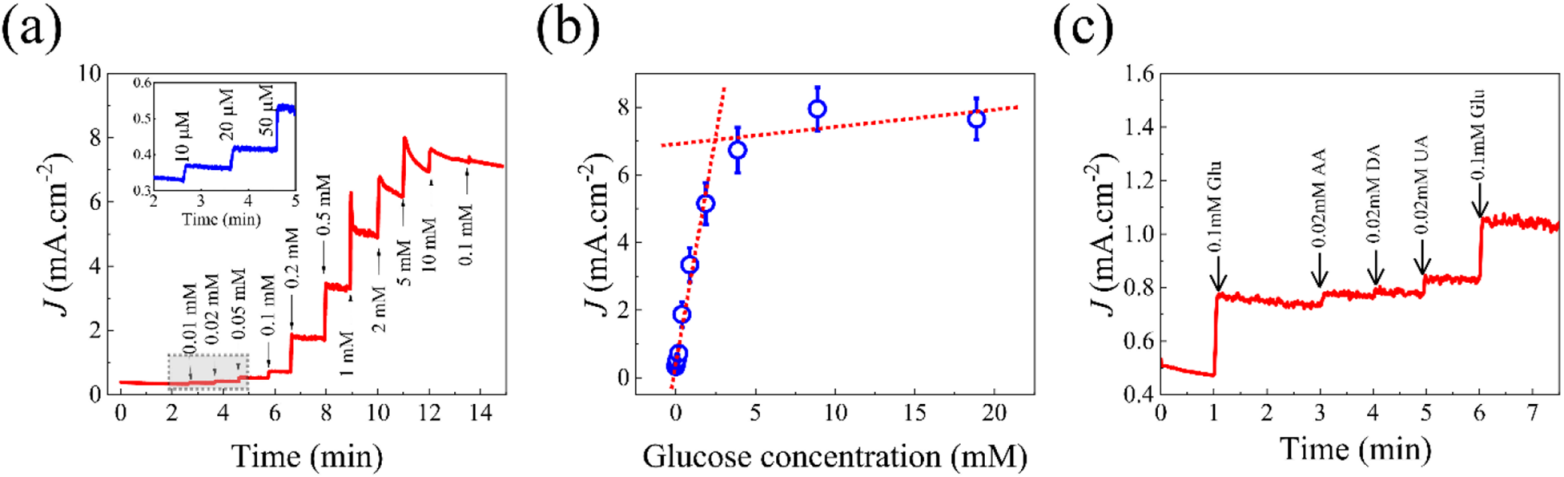
Amperometric study of Cu–Ni electrode: (**a**) Amperometric response of the Cu–Ni thin film electrode to successive addition of glucose in 0.1 M NaOH at an applied potential of 0.65 V, (**b**) corresponding calibration curve, and (**c**) amperometric response of the Cu–Ni thin film electrode to interferences AA, DA and UA.

The amperometric measurements of Cu–Ni thin film electrode is displayed in Fig. 3a. The results were obtained at 0.65 V potential in 0.1 M NaOH with increasing concentration of glucose (0.01–10 mM, with 0.01, 0.02, 0.05, 0.1, 0.2, 0.5, 1, 2, 5 and 10 mM successive injections). The Cu–Ni thin film electrode exhibited a rapid response when glucose was introduced and the current saturated within 3–5 s. The inset of Fig. 3a displayed a magnified version of amperometric curve at lower concentrations which clearly shows a significant amount of current increase with the addition of 10 µM. The linear correlation (*J* (mA cm^−2^) = (2.67219 ± 0.19054) × C (mM) + (0.44284 ± 0.1428)) between glucose concentration and the current was displayed in Fig. 3b. The respective correlation coefficients (*R*^2^) were high, 0.9704, at a lower concentration. The amperometric measurements of Cu and Ni thin film electrodes are presented in Figs. S8a and S9a and respective calibration plots presented in Figs. S8b and S9b. A wide linear range from 0.01 to 2.0 mM for glucose sensing behavior was observed for Cu–Ni thin film. In addition, the detection limit (LOD) of Cu–Ni thin film electrode was 1.86 μM which is 13 times lower than the Cu electrode and 50 times lower than Ni electrode and a high sensitivity 3517.2 μA mM^−1^ cm^−2^ which is 2.2 times greater than Cu and 19 times greater than Ni electrodes was achieved. This result shows that the proposed sensor has excellent sensitivity and very low detection limit. A composite material made of Ni–Cu/CNT/FTO^39^ for non-enzymatic glucose sensor displayed a linear range between 0.02 and 4.5 mM, a detection limit of 2 μM, and a sensitivity of 1836.5 μA mM^−1^ cm^−2^. Additionally, the electro-analytical performance of Cu–Ni electrode was compared with other Cu- and Ni-based catalysts tested in NaOH for glucose sensing as summarized in Table 1. Our Cu–Ni thin film electrode exhibit competitive performance with high sensitivity (3517.2 μA mM^−1^ cm^−2^) and a low detection limit (1.86 μM). This performance can be attributed to the synergistic activity of Cu and Ni atoms and the presence of a high density of grain boundaries (electrochemically active sites).

**Table 1:** Comparison of the glucose sensing characteristic of Cu–Ni thin film electrode with other Cu and Ni based electrodes.

Sample	Working potential (V)	Detection limit (μM)	Sensitivity (µA mM^–1^ cm^–2^)	Linear range (mM)	Ref.
Cu/G	0.4	2.47	145.52	0.01–0.2	^41^
Cu_2_O/GWs/CFP	0.55	0.21		0.0005–5	^39^
Cu_2_O/PtE	0.55	26	507	2.5	^36^
CuNPs/GP/GCE	0.5	0.2	607	0.005–1.4	^42^
Cu–CuO nanowire	0.3	500	NA	0.3–12	^43^
CuO nanorods-graphite	0.6	4	371.4	8	^44^
CuO nanospheres	0.6	1	404.5	2.6	^45^
CuO nanoplatelets	0.55	0.21	3490.7	3.5	^46^
Cu nanocluster-CNTs	0.65	0.2	251.4	2.5	^47^
NiO/GNS/GCE	0.5	0.2		4.2	^48^
Ni/NiO-Nafon-rGO/SPE	0.55	1.8		6.4	^49^
NiO nanosheet		2	3400	0.18	^50^
Ni–Cu/CNT/GCE		0.025	2633	0.025–800	^51^
Ni–Cu/CNT/FTO		2	1836.5	20–4500	^38^
Cu	0.65	23.4	1574.1	0.01–2.0	This work
Ni	0.65	91.67	182.6	0.01–2.0	This work
Cu–Ni	0.65	1.86	3517.2	0.01–2.0	This work

It is most important for a biosensor to discriminate against electroactive interfering species. In typical physiological sample, interfering species, such as ascorbic acid (AA), dopamine (DA), uric acid (UA) co-exists along with the glucose^41^. However, the glucose concentration (3–8 mM) is significantly higher in comparison to the concentration of AA (~ 23 µM), UA (0.13–0.46 mM) and DA (~ 0.1 mM) in normal conditions. Therefore, we performed CA experiments in the presence of 0.1 mM glucose and 0.02 mM of each interreference. The addition of glucose results in an immediate and significant increase in the glucose oxidation current. As shown in Fig. 3c, the Cu–Ni thin film catalyst displays excellent selectivity for glucose oxidation; the corresponding oxidation current change is 270 μA cm^−2^ upon adding 0.1 mM glucose, which greatly exceeds those recorded for the 0.02 mM concentration of interfering species, i.e., 33 μA cm^−2^ for AA, 12 μA cm^−2^ for DA and 45 μA cm^−2^ for UA. The signal for glucose oxidation overshadowed the response for interreference despite high working potential resulting in higher current. Considering that in case of diabetic condition, glucose concentration in human blood samples is much higher than in the normal case. The obtained slight response for the interference will be negligible. These results suggest that Cu–Ni electrodes exhibit a reliable anti-interference characteristic and they can be used for practical biotechnology applications. For comparison, the effect of interferences on Cu (Fig. S10a) and Ni thin film (Fig. S10b) electrodes are presented in supplementary information.

### Reproducibility and long-term stability of Cu–Ni electrodes

**Figure 4:**
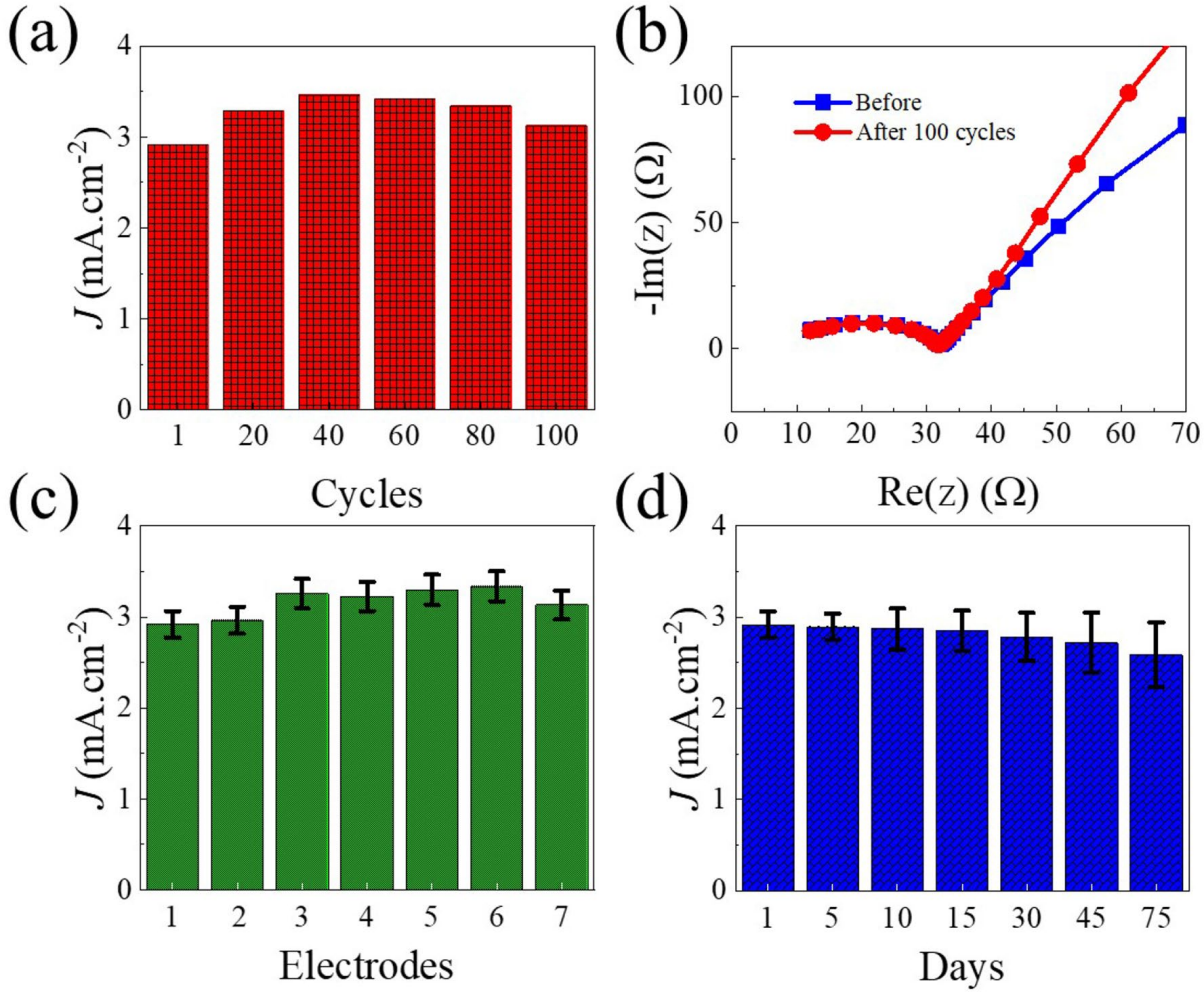
Durability, repeatability and stability of Cu–Ni electrodes: (**a**) Current density responds to the Cu–Ni thin film electrode for 100 cycles recorded after 20 cycles alternatively. (**b**), EIS spectra for Cu–Ni electrode before and after 100 cycles. (**c**) Current density recorded for different electrodes prepared from one wafer and (**d**) Current density responses recorded at 5 days of interval for 75 days. All experiments were performed in 1 mM glucose in 0.1 M NaOH electrolyte.

A non-enzymatic glucose sensor must show excellent reproducibility, repeatability, and long-term stability. The reproducibility of the Cu–Ni sensors was examined by performing CV for eight different electrodes in 1 mM Glucose 0.1 M NaOH solution. We exposed the Cu–Ni electrode to continuous 100 CV cycles in the range of − 0.5 to 1.0 V and in the presence of 1 mM glucose. Figure 4a shows the recorded current at 0.65 V after 20 cycles alternatively. Initially, the glucose oxidation current increases after the first cycle and attains maximum value after 40 cycles, and afterward the current slightly decreases. This phenomenon can be attributed to the formation of new active sites as a result of oxidation/reduction process. After 100 cycles, the measured peak current density remains higher in comparison to the initial current recorded for the first cycle confirming the stability and reproducibility of the Cu–Ni electrodes. Moreover, electrochemical impedance spectroscopy (EIS) spectra were also recorded before and after 100 cycles (Figs. 4b, S11). The similar charge transfer resistance (~ 26 Ω) with other circuit components confirmed the stability of the electrode. Seven different electrodes from the same wafer were tested to explore the reproducibility of electrodes (Fig. 4c). The results confirm that there is slight variation (~ 7%) from one sample to another suggesting homogeneity of the film and reproducibility of the electrodes. The repeatability was also examined by testing the glucose sensing performance of individual Cu–Ni electrode for 75 days. The recorded current and shape of the CVs remain identical confirming the excellent repeatability of the Cu–Ni sensors. While the sample was stored at room temperature, it shows excellent durability for the examined period (75 days) with only 10% drop in activity after this duration (Fig. 4d).

## Conclusion

In summary, a wafer-scale and chemical-free Cu–Ni electrodes were prepared via RF co-sputtering deposition method. The prepared Cu–Ni thin film was used as an electrode for non-enzymatic glucose biosensors. The Cu–Ni biosensors exhibits remarkable high sensitivity (3517.2 μA mM^−1^ cm^−2^), wide linear range (0.01 mM – 2 mM) and low detection limit (1.86 μM). More importantly, the results showed that the improved performance of the Cu–Ni electrode is due to the synergistic effect of the bimetallic Cu–Ni. The Cu–Ni electrodes also exhibit excellent anti-interference characteristic essential for glucose detection in biological fluids. Wafer-scale and chemical-free growth of Cu–Ni thin film electrodes resulted in the development of a non-enzymatic glucose sensor that is reproducible and reusable as evidenced by sustainable performance for 100 cycles. The Cu–Ni electrode also displayed robust performance with a slight degradation (~ 12%) after storage at ambient temperature for 75 days. Due to the industrial compatible Cu–Ni thin film growth process and its excellent performance, Cu–Ni film can be considered an excellent candidate for fabricating glucose biosensors in clinical and biotechnology fields.

## Experimental methods

### Chemical reagents

Sodium hydroxide (NaOH, pellets) purchased from Millipore Corp. Glucose (C_6_H_12_O_6_, 99%) purchased from Millipore Sigma, Uric acid (C_5_H_4_N_4_O_3_, 99%) and dopamine hydrochloride (C_8_H_11_NO_2_·HCl, 99%) purchased from Alfa Aesar. l-ascorbic acid (C_6_H_8_O_6_, 99%) purchased from Acrōs Organics. All reagents were used as received without further purification.

### Thin film deposition

Nickel (Ni) and copper–nickel (Cu–Ni) thin films were prepared by RF magnetron sputtering technique with Ni and Cu targets (50 mm in diameter, 99.99% purchased from Kurt J. Leskar). A schematic of the sputtering deposition chamber is displayed in Fig. 1a. The deposition chamber was pumped down to 10^−8^ Torr and filled with argon gas maintained at 10 mTorr. 50 W power was applied to the sputter gun and deposited plasma onto a rotating substrate. Power was applied to only Ni target for Ni thin film growth; power was applied to Ni and Cu targets simultaneously to deposit Cu–Ni co-sputtered thin film.

### Material characterization

The morphological structure and elemental composition of the samples were characterized using a scanning electron microscopy (SEM). Surface morphologies of sputter-deposited Ni and Cu–Ni thin film samples were imaged using JEOL JSM-6060LV and JEOL JSM-6010PLUS/LA SEM. Chemical analysis was carried out using a Thermo Scientific Ultradry EDS detector attached to the SEM.

### Glucose sensing experiments

Electrochemical biosensor characterization of Ni and Cu–Ni thin films was done using BioLogic SP-200 Potentiostat. Sputter deposited Ni and Cu–Ni thin film substrates were cut into 1 cm × 1.5 cm dimensions and used as the working electrode. The working electrode was attached onto a wafer holder and submerged 1 cm deep into the 0.1 M NaOH electrolyte for electrochemical measurements. CV and chronoamperometry (CA) experiments were performed on bulk Cu, sputter-deposited Ni and co-sputtered Cu–Ni thin film samples as working electrodes, Ag/AgCl (saturated KCl) as reference electrode, and Pt mesh as the counter electrode in 50 mL of 0.1 M NaOH stirring at 250 rpm at room temperature.

## Supplementary Information

Supplementary Figures.

Supplementary Information 2.

## Data Availability

All data generated or analyzed during this study are included in this published article (and its Supplementary Information files).
