# Supplementary material for: Cu and Ni Co-sputtered heteroatomic thin film for enhanced nonenzymatic glucose detection
Source: Sci Rep. 2022 May 7;12:7507. doi: 10.1038/s41598-022-11563-4 (PMC9079054; doi:10.1038/s41598-022-11563-4)
Supplement: Supplementary file 1 — Supplementary Figures. [file 41598_2022_11563_MOESM1_ESM.docx]

**Cu and Ni co-sputtered Heteroatomic Thin Film for Enhanced Nonenzymatic Glucose Detection**

Brianna Barbee^1^, Baleeswaraiah Muchharla^1^, Adetayo Adedeji^2^, Abdennaceur Karoui^3^, Kishor Kumar Sadasivuni^4*^, Mizaj Shabil Sha^4^, Aboubakr M. Abdullah^4^, Gymama Slaughter^5^, and Bijandra Kumar^1*^

^1^Department of Mathematics, Computer Science and Engineering Technology, Elizabeth City State University, Elizabeth City, NC 27909 USA.

^2^Department of Natural Sciences, Elizabeth City State University, Elizabeth City, NC 27909 USA.

^3^Center for Research Excellence in Science and Technology (CREST), Department of Mathematics and Physics, North Carolina Central University, Durham, NC 27707 USA.

^4^Center for Advanced Materials, Qatar University, Doha 2713, Qatar.

^5^Center for Bioelectronics, Old Dominion University, 4211 Monarch way, Norfolk, VA 23508, USA.

**Fig S1.** CV of Cu electrode in the presence of 1mM glucose concentration compared with CV of no glucose concentration.

**Fig S2.** CV of Ni thin film electrode in the presence of 1mM glucose concentration compared with CV of no glucose concentration

**Fig S3.** CVs of Cu electrode at different scan rates from 5 mV s^−1^ to 200 mV s^−1^ in 0.1 M NaOH with 1.0 mM glucose on scan rate

**Fig S4.** CVs of Ni electrode at different scan rates from 5 mV s^−1^ to 200 mV s^−1^ in 0.1 M NaOH with 1.0 mM glucose on scan rate

**Fig S5.** glucose oxidation current versus square root of scan rate of Cu electrode fitted with straight line and inset shows oxidation current versus scan rate for the comparison.

**Fig S6.** glucose oxidation current versus square root of scan rate of Ni thin film electrode fitted with straight line and inset shows oxidation current versus scan rate for the comparison.


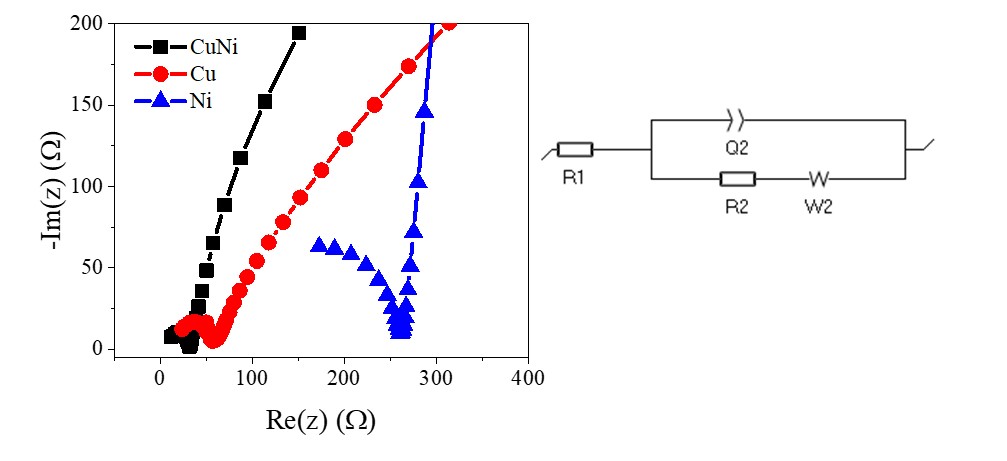


**Fig S7.** Nyquist plots of Cu, Ni thin film and Cu-Ni thin film electrodes and equivalent circuit.

Table S1. Electrochemical impedance spectroscopy (EIS) parameters of Cu, Ni and Cu-Ni thin film electrodes

| **Sample** | **R1 (Ω)** | **R2 (Ω)** |
| --- | --- | --- |
| Cu | 12.38 | 47.37 |
| Ni | 50 | 212.7 |
| Cu-Ni | 6 | 26.15 |

**Fig S8.** (a) Amperometric response of the Cu electrode to successive addition of glucose in 0.1M NaOH at an applied potential of 0.65 V, (b) corresponding calibration curve. Linear regression at the low glucose concentration region is expressed as J (mA.cm^-2^) = (1.57408 ± 0.08852) * C (mM) + (0.11855 ± 0.06634)

**Fig S9.** (a) Amperometric response of the Ni thin film electrode to successive addition of glucose in 0.1M NaOH at an applied potential of 0.65 V, (b) corresponding calibration curve. Linear regression at the low glucose concentration region is expressed as J (mA.cm^-2^) = (0.18391 ± 0.01579) * C (mM) + (0.24365 ± 0.01184).

**Fig S10.** Amperometric responses of (a) Cu electrode and (b) Ni thin film electrode to interferences AA, DA and UA.


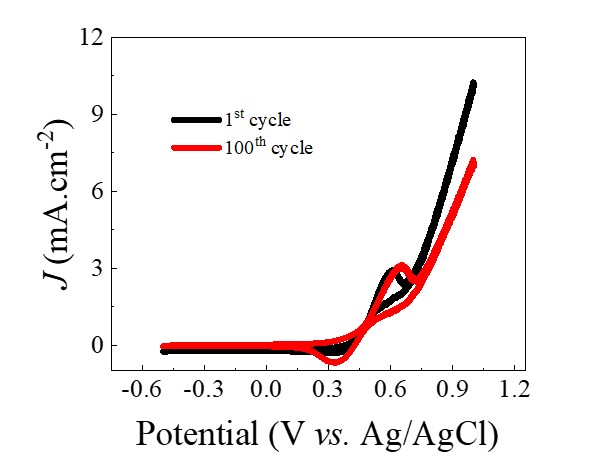


**Fig S11.** CVs of Cu-Ni thin film electrode before and after 100 cycles.
